# Management of critical-sized bone defects in the treatment of fracture-related infection: a systematic review and pooled analysis

**DOI:** 10.1007/s00402-020-03525-0

**Published:** 2020-08-29

**Authors:** H. Bezstarosti, W. J. Metsemakers, E. M. M. van Lieshout, L. W. Voskamp, K. Kortram, M. A. McNally, L. C. Marais, M. H. J. Verhofstad

**Affiliations:** 1 Trauma Research Unit, Department of Surgery, Erasmus MC, University Medical Center Rotterdam, P.O. Box 2040, 3000 CA Rotterdam, The Netherlands; 2 Department of Trauma Surgery, University Hospitals Leuven, Louvain, Belgium; 3 Nuffield Orthopaedic Centre, Oxford University Hospitals, Oxford, UK; 4 Department of Orthopaedics, School of Clinical Medicine, University of KwaZulu-Natal, Durban, South Africa; 5 Department of Development and Regeneration, KU Leuven, Louvain, Belgium

**Keywords:** Fracture-related infection, Bone transport, Induced membrane technique, Vascularized bone graft, Treatment

## Abstract

**Purpose:**

This systematic review determined the reported treatment strategies, their individual success rates, and other outcome parameters in the management of critical-sized bone defects in fracture-related infection (FRI) patients between 1990 and 2018.

**Methods:**

A systematic literature search on treatment and outcome of critical-sized bone defects in FRI was performed. Treatment strategies identified were, autologous cancellous grafts, autologous cancellous grafts combined with local antibiotics, the induced membrane technique, vascularized grafts, Ilizarov bone transport, and bone transport combined with local antibiotics. Outcomes were bone healing and infection eradication after primary surgical protocol and recurrence of FRI and amputations at the end of study period.

**Results:**

Fifty studies were included, describing 1530 patients, the tibia was affected in 82%. Mean age was 40 years (range 6–80), with predominantly male subjects (79%). Mean duration of infection was 17 months (range 1–624) and mean follow-up 51 months (range 6–126). After initial protocolized treatment, FRI was cured in 83% (95% CI 79–87) of all cases, increasing to 94% (95% CI 92–96) at the end of each individual study. Recurrence of infection was seen in 8% (95% CI 6–11) and amputation in 3% (95% CI 2–3). Final outcomes overlapped across treatment strategies.

**Conclusion:**

Results should be interpreted with caution due to the retrospective and observational design of most studies, the lack of clear classification systems, incomplete data reports, potential underreporting of adverse outcomes, and heterogeneity in patient series. A consensus on classification, treatment protocols, and outcome is needed to improve reliability of future studies.

## Introduction

Segmental bone loss after trauma remains a challenging problem for orthopedic trauma surgeons. When a bone defect exists combined with fracture-related infection (FRI), the chances of successful bone consolidation and clearance of infection are reduced.

A ‘critical-sized’ defect is a bone defect which is not expected to heal in the absence of a secondary (surgical) intervention. There is no agreed definition of what constitutes a critical defect in humans. Court-Brown defined it [1] as a defect involving 50% of the cortical diameter with a minimum length of 1 cm, and this was used in the Study to Prospectively evaluate Intramedullary Nails in Tibial fractures (SPRINT) [2]. A study by Sanders et al. [3] showed that, when using this definition, 47% of the bone defects healed without additional surgery, thus indicating that these are not always critical defects. When infection is present, it is much less likely that the fracture will heal [4, 5], so in this analysis, The SPRINT definition for a critical-sized defect in FRI was accepted.

Over the past decades, critical-sized bone defects in FRI have been treated using different protocols. Techniques used (e.g., Ilizarov, Papineau, Masquelet, or RIA) all have different indications and success rates, resulting in a wide range of clinical outcomes. The aim of this systematic review and pooled analysis was to evaluate identified treatment strategies, their individual success rates and other outcome parameters regarding critical-sized bone defects used between 1990 and 2018.

## Methods

### Literature search strategy

A literature search was completed with the help of a biomedical information specialist on June 25, 2018, using Medline, Embase, Web of Science, Cochrane, and Google Scholar. The search strings are provided in Appendix 1. Studies that described treatment of FRI using autologous cancellous grafts, autologous cancellous grafts combined with local antibiotics, the induced membrane technique, vascularized grafts, bone transport, and bone transport combined with local antibiotics were included. Series needed to be greater than five patients, reported in English, and bone defects described as ≥ 1 cm. Studies that did not describe FRI patient treatment and publications reporting non-original data (e.g., reviews or meta-analyses), or those published before 1990, were excluded. Inclusion was agreed by two independent reviewers, HB an LWV, and consisted of two phases. During the first phase, title and abstract were screened for relevance and full text articles were obtained. When possible, full texts that were not available, were obtained by contacting the corresponding author once by email. The second phase consisted of reviewing the full text articles. Consensus was reached on all references. This study was performed using the Preferred Reporting Items for Systematic Reviews and Meta-Analyses (PRISMA) criteria [6].

### Data extraction

Data from each included study were extracted by two authors independently (HB an LWV). Disagreements were discussed until agreement was reached. Data were collected in three areas.

1. General information of all studies (i.e., sample size, age, FRI, and location of FRI).
2. Data from surgical protocols (i.e., number of stages in surgical protocol, bone defect size, type of bone graft and type of fixation used).
3. Clinical outcomes (bony consolidation without infection after the primary surgical protocol, bony consolidation without infection after the study period, recurrence of FRI, amputation of the affected limb, number of complications, revision surgery, time to bony union, and Length of Hospital Stay (LOHS).

### Analysis

A quality assessment of all included studies was done according to the revised and validated versions of MINORS [7]. Results for the total population were pooled and presented separately for each of the six different treatment strategies. Medcalc (MedCalc Statistical Software version 17.9.7, MedCalc Software bvba, Ostend, Belgium; http://www.medcalc.org; 2017) was used for pooling binominal data. Heterogeneity was quantified using the Cochran’s *Q* test and *I*^2^ statistic. When *I*^2^ was < 40%, a fixed effects model was used, and a random effects model, when *I*^2^ was ≥ 40%. Pooled estimates are reported with their 95% confidence intervals (CI). Publication bias was assessed from funnel plots for each clinical outcome and per treatment type separately. The majority of studies only provided a mean but not the standard deviation. Thus, a full meta-analysis for continuous data was not feasible. Continuous data were pooled by calculating the weighted mean using Microsoft Excel. Sample size of the individual studies was used as weighting factor. The pooled mean is reported with the range.

## Results

### General population demographics

**Fig. 1:**
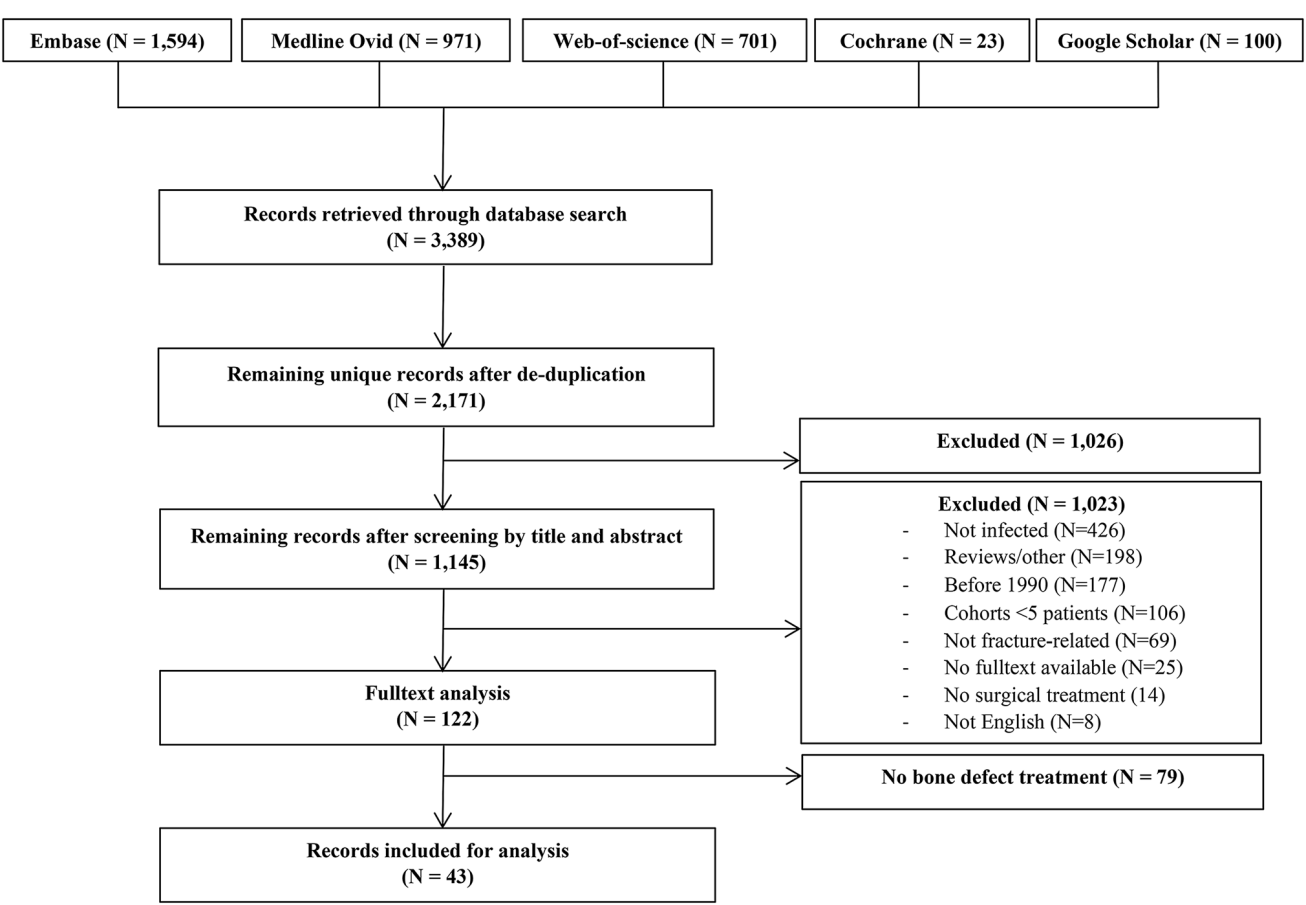
Inclusion flowchart

After selection, 43 studies [8–50] were included describing 50 patient series in the treatment of FRI with bone defects of ≥ 1 cm (Fig. 1). In these studies, 1530 patients with FRI were treated, with a mean bone defect of 6.6 cm (range 1.0–26.0), of which 1253 (82%) were localized at the tibia. The population had a mean age of 40 years (range 6–80), with 1176 (79%) male patients and a mean duration of infection of 17 months (range 1–624). Mean follow-up was 51 months (range 6–126). Study characteristics and quality assessment according to the MINORS score are given in appendix 2. The type of bone grafts used are depicted in Table 1.

**Table 1:** Bone grafts used in treatment of bone defects

Type used in total (50 patient series)	*N* = 1063 (% of total)
Non-vascularized cancellous bone	639 (60%)
Iliac crest	600 (56%)
With granulocytes	139
With BMP-7	10
RIA	39 (4%)
With BMP-7	37
Vascularized bone	324 (30%)
Fibula	260 (25%)
Latissimus Dorsi with rib	41 (4%)
Ilium	18 (2%)
Scapula	5 (1%)
Other	
Mesenchymal stem cells	15 (1%)
Graft not defined	70 (6%)

*BMP* bone morphogenetic protein, *N* total number of bone grafts used, *RIA* reamer irrigator aspirator

Nine series (18%) described treatment of bone defects by cancellous grafts alone and nine series (18%) by the use of a cancellous graft in combination with local antibiotics, either beads or an antibiotic rod. The induced membrane technique was described in eight (16%) series, and treatment with a vascularized bone graft was used in four series (8%). Bone transport alone was used in 15 series (30%), and bone transport in combination with local antibiotic beads in 5 (10%).

### Description by treatment type

Table 2 summarizes the population of all treatment types and Table 3 explains all different surgical stages.

**Table 2:** Study characteristics

Overall	Series (*N*)	Patients (*N*)	Age (years)	FU (months)	Defect size (cm)
	50	1530	40 (6–80)	51 (6–126)	6.6 (1–26)
Surgical protocol					
Cancellous graft only	9	311	40 (16–68)	61 (10–120)	4.6 (1–16)
Cancellous graft with AB	9	161	37 (18–79)	43 (24–126)	4.9 (1–12)
Induced membrane	8	177	42 (16–72)	26 (13–72)	4.5 (1–26)
Vascularized graft	4	322	43 (6–69)	75 (6–86)	10.7 (1–21)
Bone transport only	15	395	37 (17–80)	34 (12–106)	5.5 (1–21)
Bone transport with AB	5	164	43 (15–68)	49 (12–102)	5.7 (3–14)

Age; *FU* follow-up; and defect size are depicted by mean (range); *AB* antibiotics

**Table 3:** Surgical stages explained

Surgical protocol (series)	Stage description [References]
Cancellous graft only [9]	
One stage [4]	Debr. CBG [15, 18, 22, 42]
Two stage [3]	Debr. delayed CBG [26, 36, 44]
Three stage [1]	Debr. delayed CBG, delayed STC [33]
Cancellous graft with AB [9]	
Two stage [6]	Debr. local AB, delayed CBG and def. fix [10, 24, 32, 34, 35, 38]
Three stage [2]	Debr. two times local AB, delayed CBG and def. fix [11]
	Debr. local AB, delayed STC, delayed CBG and def. fix [49]
Induced membrane [8]	
Two stage [8]	Debr. local AB, delayed CBG and def. fix. [23, 29, 30, 34, 37, 40, 43, 48]
Vascularized graft [4]	
One stage [2]	Debr. VBG [16, 46]
Two stage [1]	Debr. local AB, VBG [41]
Bone transport only [15]	
One stage [12]	Debr. Ilizarov application [8, 14, 25–28, 36, 38, 39, 47, 50]
Three stage [1]	Debr. delayed tibia osteotomy, arthrodesis ankle [12]
Bone transport with AB [5]	
One stage [1]	Debr. CaSO4 AB pellets, Ilizarov application [40]
Two stage [4]	Debr. local AB, delayed Ilizarov application [13, 18, 20, 31]
Unspecified [5]	[9, 17, 19, 21, 45]

*AB* antibiotics, *Debr* debridement, *CBG* cancellous bone graft, *STC* soft tissue coverage, *AB* antibiotics, *def. fix.* definitive fixation, *VBG* vascularized bone graft

#### Cancellous grafts (with or without local antibiotics)

In 18 studies [9–11, 15, 17, 18, 22, 24, 26, 32–36, 38, 42, 44, 49] (36%), a total of 472 (31%) patients were treated with a cancellous bone graft, half of these studies used a cancellous graft only, the other half used a cancellous bone graft after local antibiotics treatment with either Polymethyl methacrylate (PMMA) beads or a cement rod.

The 9 studies (18%) describing treatment with a cancellous graft only included 311 (20%) patients, with a mean age of 40 years (range 16–68) and a mean bone defect of 4.6 cm (range 1–16). Four studies (44%), used a one-stage reconstruction, three studies (33%) a two-stage, and one study (11%) a three-stage. One study (11%) did not describe the number of stages.

The other 9 studies (18%), which used a cancellous graft after placement of PMMA beads or a cement rod, described the treatment of 161 (11%) patients. The mean patient age was 37 years (range 18–79) and the mean bone defect length was 4.9 cm (range 1–12). The majority, 67%, were treated by a two-stage protocol, two studies (22%) used a three-stage protocol and one (11%) did not mention the number of stages. Mean time between placement and removal of the antibiotic carrier was 31 days.

#### Induced membrane technique

Eight series [23, 29, 30, 34, 37, 40, 43, 48] (16%) described the treatment of 177 (12%) patients, with a mean age of 42 years (range 16–72), a mean bone defect size of 4.5 cm (range 1.0–26.0), that were followed-up for a mean of 26 months (range 13–72). All eight studies used a two-stage reconstruction protocol with an antibiotic loaded cement spacer, which was removed after a mean time of 68 days.

#### Vascularized grafts

In four (8%) studies [16, 21, 41, 46], a total of 322 (21%) patients were treated with a vascularized bone graft all using mixed, internal and external, fixation protocols. Two studies (50%) used a single-stage procedure, one study (25%) a two-stage procedure, and one study (25%) did not describe the number of stages. Their mean patient age was 43 years (range 6–69), with a mean defect size of 10.7 cm (range 1.0–21.0), and a mean follow-up of 75 months (range 6–86).

#### Bone transport

In 20 studies [8, 12–14, 18–20, 25–28, 31, 36, 38–40, 45, 47, 50] (40%), 559 (37%) patients were treated using a bone transport technique. In 15 (75%) of these studies, bone transport was used without the use of local antibiotic therapy. These series described the treatment of 395 (26%) patients with FRI, with a mean age of 37 years (range 17–80), and a mean defect length of 5.5 cm (range 1–21). Twelve studies (80%) mentioned a one-stage bone transport procedure, one (2%) described a three-stage bone transport procedure with open wound treatment, and two (4%) did not mention the number of stages.

The remaining five studies (26%) used a combination of bone transport and local antibiotics to treat 164 (11%) patients, aged 43 years (range 15–68), with a mean bone defect of 5.7 cm (range 3–14). All studies used local antibiotics in the form of beads, four studies (80%) used PMMA as a carrier in a two-stage design, and one (20%) used calcium sulphate pellets in a one-stage protocol. Mean time before removal of the PMMA beads was 42 days.

### Clinical outcome of all studies

After initial protocolized treatment, FRI was cured and bone defects healed in 83% (95% CI 79–87) of all cases, increasing to 94% (95% CI 92–96) with further treatment, at the end of the total study period. Recurrence of infection was seen in 8% (95% CI 6–11), and amputation in 3% (95% CI 2–3) of all cases.

**Fig. 2:**
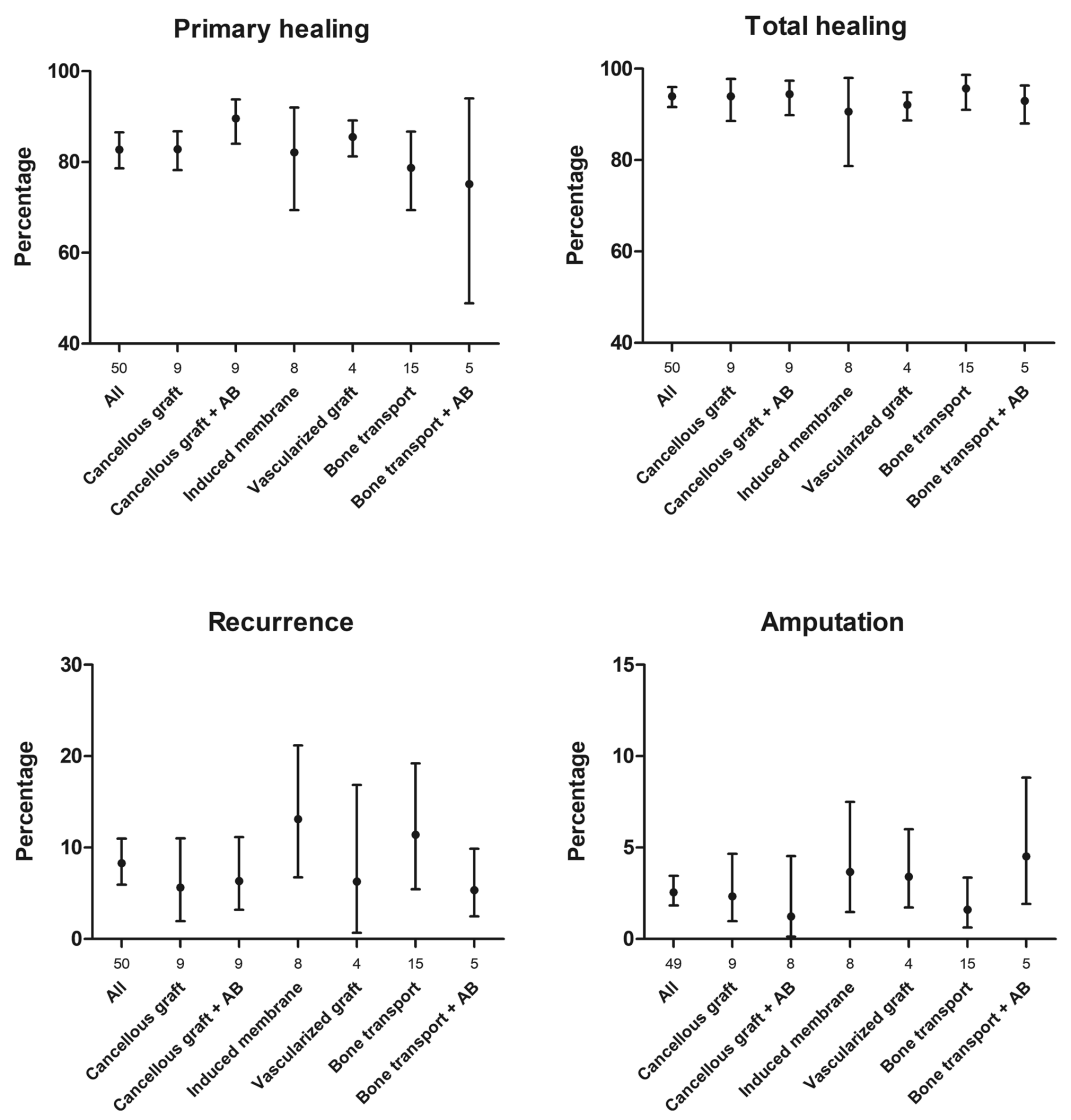
Proportional clinical outcome. Numbers under the *X*-axis represent the number of studies for which data are pooled; *AB* local antibiotics

Figure 2 shows the clinical outcome for all studies. Final outcomes overlapped across all different treatments. The use of the induced membrane or bone transport technique shows a trend of increased recurrence of infection when compared to the other techniques. Reconstruction with the induced membrane technique, a vascularized graft, or bone transport with local antibiotics tends to lead to a larger amputation rate than the bone transport and two other cancellous graft treatments alone. Funnel plots assessing publication bias for each clinical outcome are presented per treatment type in Appendix 3. The funnel plots of primary and total healing both show comparable asymmetry, possibly due the smaller studies having worse outcome. Publication bias could have occurred because of different study size populations in mostly retrospective series underreporting negative outcomes. This could also be the case for the funnel plots depicting recurrence of infection and amputation ratio. Half of all studies do not report recurrence of infection, possibly due to better surgical protocols, or underreporting of negative clinical outcome in mostly retrospective series. Forest plots depicting clinical outcome per treatment type are given in Appendix 4.

**Fig. 3:**
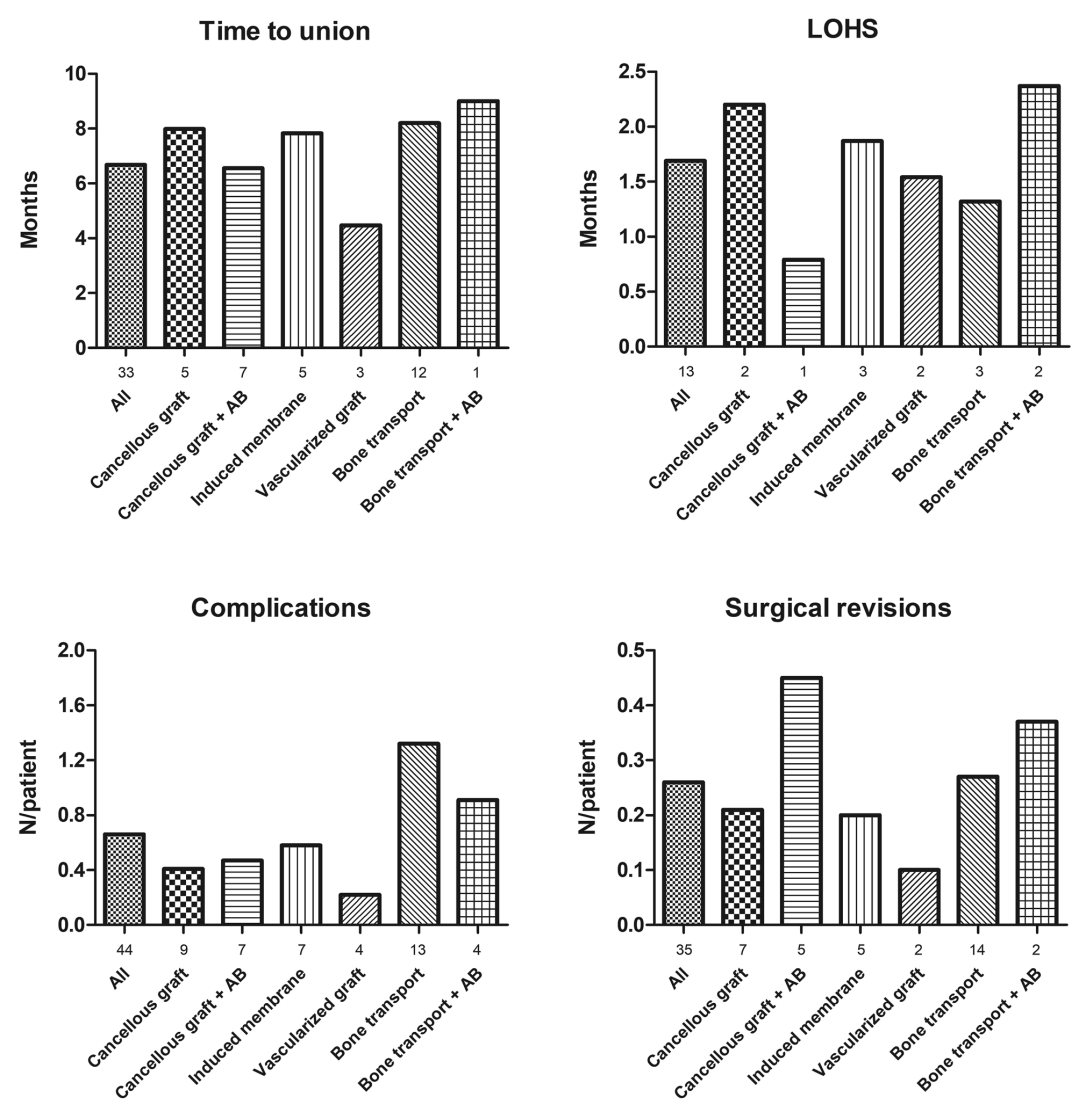
Clinical outcome by weighted mean. Numbers under the *X*-axis represent the number of studies for which data are pooled; *AB* local antibiotics

Figure 3 shows the weighted means for all continuous outcomes. In 33 (66%) studies reporting time to union, bony union was achieved after a mean of 6.7 months. Patients were hospitalized for a mean of 1.7 months. A mean of 0.7 complications per patients were recorded, i.e. superficial or deep infection, hemorrhage, deformities, and non-union. Almost one-third of all patients required at least one reoperation. Overall time to union, LOHS, complication rate per patient, and surgical revisions per patient could not be compared between groups. The use of vascularized grafts tends to have a shorter time of union, number of complications and less necessary surgical revisions when compared to the other groups.

## Discussion

Adequate debridement of non-viable bone remains one of the cornerstones of the management of FRI [51, 52]. This debridement, along with the bone loss that occurs at the time of the initial trauma and due to the infectious process itself, frequently results in the presence of significant bone defects in patients with FRI. The optimal management of these bone defects remains controversial, leading to the numerous techniques described [53].

This systematic review revealed that the current most popular techniques resulted in comparable rates of healing and recurrence of infection. It is however unlikely that one reconstruction technique will be able to address all types of bone defects in FRI patients. Kadhim et al. [54] reviewed the use of bone transport or a vascularized bone graft for reconstruction of segmental bone defects, showing different success rates at different anatomical locations. Other recent reports have raised concern with regards to the efficacy of the induced membrane technique, for example, as a reconstruction technique of post-infective tibial shaft defects [30, 55, 56]. The need therefore is to determine what technique would be deemed optimal in a certain specific clinical scenario.

Due to several factors, it was not possible to draw firm conclusions from the data in this regard. Firstly, the heterogeneity of the patient populations made comparison of outcomes difficult. There was, for example, a difference in the mean size of the defects among the different treatment groups. Series describing the use of vascularized grafts or bone transport techniques treated larger bone defects than series using cancellous grafts. Also, in studies describing a two-stage reconstruction protocol, often more surgical revisions were performed than initially expected.

The choice of reconstruction technique depends on many factors. These include the host’s physiological status and ability to participate in the rehabilitation program, the shape and location of the defect, duration of the defect (i.e., acute/early or chronic/late-onset), quality of the surrounding soft tissue, bone quality, the presence of deformity, adjacent joint contracture/instability or limb length discrepancy, as well as the experience of the surgeon [57]. Unfortunately, the published series do not always report the details of these factors. It is often impossible to understand how patients were selected for a particular technique. This deficit makes comparison of outcomes difficult. The outcome of the use of one technique in compromised hosts with tibial defects, for example, cannot be compared with the results of another technique in optimized hosts in the upper limb.

Most studies provide a cohort of cases treated with a single technique, rather than a consecutive series of patients, selected because of the characteristics of the disease [58]. This makes firm conclusions on the efficacy of a technique in a patient population impossible [59].

Secondly, of all included series, 12 (24%) [20, 23, 32, 33, 35, 37, 40, 41, 43, 46, 47] describe FRI on multiple anatomical locations, and six (12%) [8, 12, 17, 38, 48] describe FRI of the femur, ankle or foot. Although these series are low in number of patients, data pooling still results in a distorted outcome.

The current lack of a universally accepted classification system for both FRI and post-infective bone defects is evident in this systematic review. The need remains for the development of a pragmatic classification system that can be used, not only to guide treatment, but also to adequately characterize the patient cohort for research purposes [60–62]. Similarly, traditional classification systems for post-infective bone defects have failed to keep up with contemporary trends in reconstruction. The classification of post-infective tibial bone defects proposed by May and Jupiter, for example, does not cater for the induced membrane technique [63].

In addition to the lack of an applicable classification of post-infective bone defects, a working definition of defects is needed which cannot be expected to heal without intervention. While the difference between a stable cavitory (contained) defect and an unstable defect may be apparent, there may be a need to further define critical unstable defects to investigate the outcomes of treatment modalities in different types of critical bone defects. This definition, together with a pragmatic classification would allow the development of a practical algorithm for management of these infected defects. It may be foreseen that a 4 cm conical (partial) humeral defect with 20% cortical bone contact may be treated differently than a 4 cm segmental (complete) tibial defect, for example [64].

In addition to the lack of uniformity in terms of patient population, numerous other factors prohibit direct comparison of results from the studies included in this systematic review. Due to the retrospective and observational nature of most studies, treatment strategy selection was not always clearly reported and may not have been consistent. Furthermore, data were not always sufficiently complete to allow comparison of outcomes. For continuous outcomes, most studies failed to report the standard deviation along with the mean value. Underreporting of adverse effects is of particular concern, especially if risks associated with a specific treatment option in a specific clinical setting should be taken into account when choosing a reconstructive option. When looking at the asymmetrical form of the funnel plots created for all clinical outcomes, this could be the case, as is discussed above.

Mauffrey and Hak [55] have recently highlighted the lack of standardization in the management algorithms for tibial defect reconstruction, particularly in the setting of post-infective defects where treatment strategy selection is often based on surgeon preference rather than scientific evidence [65]. Similarly, Makridis et al. [65] found great heterogeneity of studies in the field of FRI, which made the development of evidenced-based management protocols difficult. The lack of comparative studies is also evident. While bone transport and the induced membrane technique remain two popular treatment options, there is no high-quality evidence indicating that one is superior to the other [66], or which patients are best suited to each technique. This systematic review confirmed that evidence to make any high-level recommendation with regard to the management of post-infective bone defects in FRI patients is currently insufficient.

It is unlikely that one single, high volume center is able to adequately study the problem of FRI. Even in such referral centers the number of patients treated in a reasonable time span is not sufficient to achieve enough statistical power to draw firm conclusions on current treatment standards and new therapeutic options. This implies that (international) collaboration is essential to be able to pool treatment results from individual hospitals into (prospective) clinical studies and subsequently into meaningful meta-analyses. Only then, can adequate progress in the treatment of FRI be made. To facilitate such a collaboration, uniform definitions for classification, diagnostic and treatment protocols and follow-up are required. The current meta-analysis clearly shows that the way in which data have been collected and reported in the past cannot inform best practice in the future.

## Conclusions

This is the first extensive review of bone defect treatment protocols for FRI. Six individual treatment protocols for FRI treatment were identified, i.e., cancellous grafts with or without the use of local antibiotics, the induced membrane technique, the use of vascularized grafts, or the Ilizarov bone transport technique with or without the use of local antibiotics. All show comparable outcome. Overall published work showed a high success rate of 94%, a recurrence rate of 8%, and low amputation rate of only 3%. However, data did not allow a reliable comparison across treatments, or a recommendation on which treatment strategy is appropriate for any particular clinical scenario. The results should thus be interpreted with caution due to the retrospective design of most studies, the lack of clear classification systems, incomplete data reports, underreporting of adverse outcomes, and heterogeneity in patient series. Secondly, this review reveals the true scientific and clinical needs: uniform definitions on terms used, a consensus on classification, structured treatment protocols, and clear outcome parameters are needed to improve reliability of future multicenter studies.

## Appendix 1: Search terms used for the individual databases

**(A) Medline OVID:** (“osteitis”/ OR “osteomyelitis”/ OR (osteitis OR osteomyelitis).ab,ti.) AND (exp “Fractures, Bone”/ OR (fracture* OR nonunion* OR malunion* OR nonunited* OR malunited* OR posttraum* OR post-traum*).ab,ti.) AND (“Surgical Procedures, Operative”/ OR “debridement”/ OR osteitis/su OR osteomyelitis/su OR (surger* OR surgic* OR debridement*).ab,ti.) AND (“observational study”/ OR exp “Cohort Studies”/ OR “Case-Control Studies”/ OR “Cross-Sectional Studies”/ OR “multicenter study”/ OR “comparative study”/ OR “clinical study”/ OR exp “clinical trial”/ OR “Random Allocation”/ OR exp “treatment outcome”/ OR (((observation* OR comparativ*) ADJ6 (stud* OR data OR research)) OR cohort* OR longitudinal* OR retrospectiv* OR prospectiv* OR ((case OR cases OR match*) ADJ3 control*) OR (cross ADJ section*) OR correlation* OR multicenter* OR multi-center* OR follow-up* OR followup* OR clinical* OR trial OR random* OR (treatment ADJ3 (outcome* OR fail* OR success*))).ab,ti.) NOT (letter OR news OR comment OR editorial OR congresses OR abstracts).pt. AND english.la.

**(B) Embase:** (‘osteitis’/de OR ‘osteomyelitis’/de OR ‘chronic osteomyelitis’/de OR (osteitis OR osteomyelitis):ab,ti) AND (‘fracture’/exp OR ‘posttraumatic complication’/de OR (fracture* OR nonunion* OR malunion* OR nonunited* OR malunited* OR posttraum* OR post-traum*):ab,ti) AND (‘surgery’/de OR ‘surgical technique’/de OR ‘debridement’/de OR ‘osteitis’/exp/dm_su OR (surger* OR surgic* OR debridement*):ab,ti) AND (‘observational study’/exp OR ‘cohort analysis’/exp OR ‘longitudinal study’/exp OR ‘retrospective study’/exp OR ‘prospective study’/exp OR ‘case control study’/de OR ‘cross-sectional study’/de OR ‘correlational study’/de OR ‘major clinical study’/de OR ‘multicenter study’/de OR ‘comparative study’/de OR ‘follow up’/de OR ‘clinical study’/de OR ‘clinical article’/de OR ‘clinical trial’/exp OR ‘randomization’/exp OR ‘intervention study’/de OR ‘open study’/de OR ‘treatment outcome’/exp OR (((observation* OR comparativ*) NEAR/6 (stud* OR data OR research)) OR cohort* OR longitudinal* OR retrospectiv* OR prospectiv* OR ((case OR cases OR match*) NEAR/3 control*) OR (cross NEXT/1 section*) OR correlation* OR multicenter* OR multi-center* OR follow-up* OR followup* OR clinical* OR trial OR random* OR (treatment NEAR/3 (outcome* OR fail* OR success*))):ab,ti) NOT ([Conference Abstract]/lim OR [Letter]/lim OR [Note]/lim OR [Editorial]/lim) AND [english]/lim.

**(C) Web of Science:** TS=(((osteitis OR osteomyelitis)) AND ((fracture* OR nonunion* OR malunion* OR nonunited* OR malunited* OR posttraum* OR post-traum*)) AND ((surger* OR surgic* OR debridement*)) AND ((((observation* OR comparativ*) NEAR/5 (stud* OR data OR research)) OR cohort* OR longitudinal* OR retrospectiv* OR prospectiv* OR ((case OR cases OR match*) NEAR/2 control*) OR (cross NEAR/1 section*) OR correlation* OR multicenter* OR multi-center* OR follow-up* OR followup* OR clinical* OR trial OR random* OR (treatment NEAR/2 (outcome* OR fail* OR success*))))) AND DT=(article) AND LA=(english).

**(D) Cochrane:** ((osteitis OR osteomyelitis):ab,ti) AND ((fracture* OR nonunion* OR malunion* OR nonunited* OR malunited* OR posttraum* OR post-traum*):ab,ti) AND ((surger* OR surgic* OR debridement*):ab,ti).

**(E) Google scholar:** “posttraumatic|traumatic osteitis|osteomyelitis” surgery|surgical|debridement allintitle:“posttraumatic|traumatic osteitis|osteomyelitis” surgery|surgical|debridement

## Appendix 2: Study characteristics and quality control

**Table:**

References	Pt (*N*)	Pct. males (%)	FRI location	Pct. tibia (%)	Surgical protocol	Type	LoE	MINORS^a^ score
Campbell et al. [9]	12	67	T	100	CG	Retrospective cohort	4	5^b^
Deng et al. [15]	15	60	T	100	CG	Retrospective cohort	4	4^b^
Eralp et al. [17]	45	84	T	100	CG	Retrospective comparison	3	11^c^
Hernigou et al. [22]	80	70	T	100	CG	Prospective comparison	2	14^c^
Marsh et al. [26]	15	87	T	100	CG	Retrospective comparison	3	9^c^
Polyzois et al. [33]	31	100	T, f	68	CG	Retrospective cohort	4	5^b^
Sadek et al. [36]	16	75	T	100	CG	Retrospective comparison	3	12^c^
Vitkus et al. [42]	29	–	T	100	CG	Retrospective cohort	4	3^b^
Wang et al. [44]	68	71	T	100	CG	Retrospective comparison	3	11^c^
Chan et al. [10]	36	83	T	100	CGAB	Retrospective cohort	4	2^b^
Chen et al. [11]	18	83	T	100	CGAB	Retrospective cohort	4	5^b^
Eralp et al. [17]	13	69	A	0	CGAB	Retrospective cohort	4	11^c^
Lin et al. [24]	16	88	T	100	CGAB	Retrospective cohort	4	2^b^
Patzakis et al. [32]	33	82	T, F, U	67	CGAB	Prospective cohort	4	8^b^
Qiu et al. [34]	18	83	T	100	CGAB	Retrospective comparison	3	11^c^
Reichert et al. [35]	14	79	T, F, R, H	29	CGAB	Retrospective cohort	4	5^b^
Shyam et al. [38]	7	86	F	0	CGAB	Prospective comparison	2	14^c^
Zalavras et al. [49]	6	83	T	100	CGAB	Retrospective cohort	4	6^b^
Jeong et al. [23]	15	67	T, A, F	47	IM	Retrospective cohort	4	5^b^
Moghaddam et al. [29]	50	84	T	100	IM	Prospective comparison	2	16^c^
Morris et al. [30]	12	75	T	100	IM	Retrospective cohort	4	4^b^
Qiu et al. [34]	22	82	T	100	IM	Retrospective comparison	3	11^c^
Scholz et al. [37]	13	92	T, f, F, R	38	IM	Retrospective cohort	4	6^b^
Tong et al. [40]	20	75	T, F	65	IM	Retrospective comparison	3	14^c^
Wang et al. [43]	32	69	T, F	63	IM	Retrospective cohort	4	7^b^
Yu et al. [48]	13	69	F	0	IM	Retrospective cohort	4	8^b^
Yang et al. [46]	51	84	T, F	92	VG	Prospective cohort	4	9^b^
Doi et al. [16]	26	85	T	100	VG	Retrospective cohort	4	3^b^
Erdinger et al. [21]	5	80	T	100	VG	Retrospective cohort	3	5^b^
Tu and Yen [41]	240	84	T, A, F	67	VG	Retrospective cohort	4	6^b^
Barbarossa et al. [8]	30	80	F	0	BT	Retrospective cohort	4	2^b^
Chen et al. [12]	12	100	A	0	BT	Retrospective cohort	4	5^b^
Dendrinos et al. [14]	28	82	T	100	BT	Retrospective cohort	4	4^b^
Eralp et al. [19]	43	91	T	100	BT	Retrospective comparison	3	9^b^
Liu et al. [25]	35	71	T	100	BT	Retrospective cohort	4	5^b^
Marsh et al. [26]	10	50	T	100	BT	Retrospective comparison	3	9^c^
McNally et al. [27]	18	–	T	100	BT	Prospective cohort	4	9^c^
McNally et al. [27]	16	–	T	100	BT	Prospective cohort	4	9^c^
Megas et al. [28]	9	78	T	100	BT	Retrospective cohort	4	5^b^
Sadek et al. [36]	14	86	T	100	BT	Retrospective comparison	3	12^c^
Shyam et al. [38]	5	100	F	0	BT	Prospective comparison	2	14^c^
Tetsworth et al. [39]	21	86	T	100	BT	Retrospective comparison	3	14^c^
Xu et al. [45]	30	70	T	100	BT	Retrospective cohort	4	6^b^
Yin et al. [47]	100	92	T, F	65	BT	Retrospective cohort	4	6^b^
Zhang et al. [50]	24	71	T	100	BT	Prospective comparison	3	9^b^
Chim et al. [13]	28	75	T	100	BTAB	Retrospective cohort	4	4^b^
Eralp et al. [20]	13	62	T, F	54	BTAB	Retrospective cohort	4	5^b^
Eralp et al. [17]	29	69	T	100	BTAB	Retrospective comparison	3	11^c^
Napora et al. [31]	75	76	T	100	BTAB	Retrospective cohort	4	5^b^
Tong et al. [40]	19	79	T, F	68	BTAB	Retrospective comparison	3	14^c^

*LoE* level of evidence, *Pct.* percentage, *Pt* patients, *FRI* fracture-related infection, *T* tibia, *F* femur, *A* ankle, *f* foot, *U* ulna, *R* radius, *H* humerus, *CG* cancellous graft, *CGAB* cancellous graft with local antibiotics, *IM* induced membrane technique, *VG* vascularized graft, *BT* bone transport, *BTAB* bone transport with local antibiotics

^a^Methodological Index for Non-Randomized Studies (7)

^b^Out of 16

^c^Out of 24
